# Chemometric Strategies for Sensitive Annotation and Validation of Anatomical Regions of Interest in Complex Imaging Mass Spectrometry Data

**DOI:** 10.1007/s13361-019-02327-y

**Published:** 2019-09-16

**Authors:** Patrick M. Wehrli, Wojciech Michno, Kaj Blennow, Henrik Zetterberg, Jörg Hanrieder

**Affiliations:** 1 Department of Psychiatry and Neurochemistry, Sahlgrenska Academy at the University of Gothenburg, Sahlgrenska University Hospital Mölndal, Mölndal, Sweden; 2 Clinical Neurochemistry Laboratory, Sahlgrenska University Hospital Mölndal, Mölndal, Sweden; 3 UK Dementia Research Institute at UCL, London, UK; 4 Department of Neurodegenerative Disease, Queen Square Instritute of Neurology, University College London, London, UK

**Keywords:** Imaging mass spectrometry (IMS), Multivariate data analysis (MVA), Data processing, Image segmentation, Regions of interest (ROIs), Active contour segmentation (ACS)

## Abstract

Imaging mass spectrometry (IMS) is a promising new chemical imaging modality that generates a large body of complex imaging data, which in turn can be approached using multivariate analysis approaches for image analysis and segmentation. Processing IMS raw data is critically important for proper data interpretation and has significant effects on the outcome of data analysis, in particular statistical modeling. Commonly, data processing methods are chosen based on rational motivations rather than comparative metrics, though no quantitative measures to assess and compare processing options have been suggested. We here present a data processing and analysis pipeline for IMS data interrogation, processing and ROI annotation, segmentation, and validation. This workflow includes (1) objective evaluation of processing methods for IMS datasets based on multivariate analysis using PCA. This was then followed by (2) ROI annotation and classification through region-based active contours (AC) segmentation based on the PCA component scores matrix. This provided class information for subsequent (3) OPLS-DA modeling to evaluate IMS data processing based on the quality metrics of their respective multivariate models and for robust quantification of ROI-specific signal localization. This workflow provides an unbiased strategy for sensitive annotation of anatomical regions of interest combined with quantitative comparison of processing procedures for multivariate analysis allowing robust ROI annotation and quantification of the associated molecular histology.

**Electronic supplementary material:**

The online version of this article (10.1007/s13361-019-02327-y) contains supplementary material, which is available to authorized users.

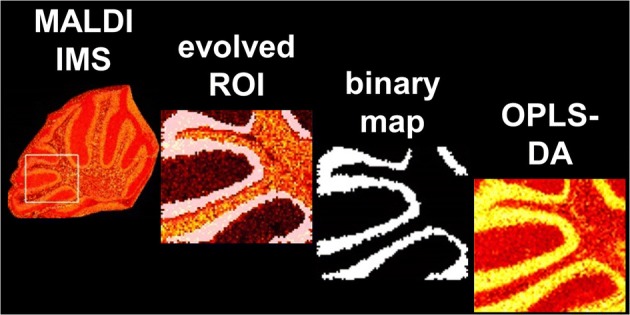

## Introduction

Imaging mass spectrometry (IMS) allows delineation of histological features based on complex chemical fingerprints. This in turn provides comprehensive insight in molecular mechanisms associated with histopathological processes [1]. These tools are now able to supplement histological staining-based assessment methods, as commonly used in pathology. IMS dramatically expands the histochemical dimensionality in pathological imaging by providing broad molecular information, about metabolites, lipids, peptides, glycans, and small proteins (for review see [2, 3]).

Multivariate tools are increasingly used to interrogate complex IMS data to identify true molecular localization in situ [4–7]. Optimal data pre-processing, including normalization and transformation, is critical to interpret IMS data and to avoid possible artifacts that may lead to misinterpretation and wrong assignment of histologically relevant chemical co-localizations [8–12]. Normalization of the IMS data is typically used to remove the systematic variation caused by non-biologically relevant artifacts, such as cutting artifacts, matrix inhomogeneity, and gradual decrease in signal associated with duration of IMS analysis. Here, total ion current (TIC), median, mean root mean square (RMS), and normalization to the maximum peak, to a various extend compensate for such artifacts. A great risk associated with these common normalization strategies is over-normalization leading to removal of weak, but biologically highly-relevant molecular signal. Several studies have evaluated the effects of data processing for interpretation and improved visualization of IMS data as well as subsequent multivariate analysis-based image segmentation [5, 10, 11, 13–15].

However, there is a high demand for unbiased strategies to evaluate IMS data processing and its effects on multivariate image analysis including objective detection of histological region of interest (ROI). In line with this, a further critical issue when analyzing IMS data is then the accurate outlining of these histological features as ROI for subsequent extraction of the associated chemical content. This ROI annotation and classification step is commonly done by outlining these ROI manually, although a variety of methods such as thresholding and clustering methods have been presented for this purpose [9, 16]. As these methods, however, all show limitations with respect to a sensitivity and accuracy, there is a need for introducing other tools for outlining ROI shapes in a straightforward and accurate manner.

We here describe a chemometric strategy for interrogation complex IMS data, to accurately identify anatomical regions of interest (ROIs) and quantify the chemical co-localization associated with these regions. For this we, evaluate IMS raw data processing and its consequences for multivariate image analysis. Here, we step outside the typical IMS processing toolbox, comprising common normalization methods (such as TIC or RMS) and processing methods (such as PCA and segmentation), and introduce conventional computational image-processing tools and advances, semi-supervised chemometric tools. Using a three-step MVA strategy involving image PCA, region-based active contour segmentation, and orthogonal projections to latent structures discriminant analysis (OPLS-DA), we quantify the performance of different preprocessing procedures, i.e., normalizations and transformations based on the quality metrics of the multivariate models.

## Experimental

### Animals and Tissue Preparation

Fresh brain tissue samples were obtained from female, 18-month-old C57BL/6 mice (*n* = 3) from Charles River Laboratories (Sulzfeld, Germany). Animals were reared ad libitum at an animal facility at Uppsala University under a 12/12 light cycle. The animals were anesthetized with isoflurane and sacrificed by decapitation. The brains were dissected quickly with less than 3 min post-mortem delay and frozen on dry ice. Animal procedures were approved by an ethical committee and performed in compliance with national and local animal care and use guidelines (DNr #C17/ 14 at Uppsala University). Frozen tissue sections (12 μm) were cut in a cryostat microtome (Leica CM 1520, Leica Biosystems, Nussloch, Germany) at − 18 °C, and collected on indium tin oxide (ITO) conductive glass slides (Bruker Daltonics, Bremen, Germany) and stored at − 80 °C. Prior to analysis, tissue sections were thawed under vacuum for 1 h.

### MALDI Imaging MS

For MALDI imaging of lipids, 1.5 di-amino-naphthalene (1,5-DAN) matrix was applied to the tissue sections using a TM sprayer (HTX Technologies, Carrboro, NC, USA) combined with a HPLC pump (Dionex P-580, Sunnyvale, CA, USA). Before spraying, the solvent pump was purged with 70% ACN at 500 μL/min for 10 min followed by manual rinse of matrix loading loop using a syringe. A matrix solution containing 20 mg/mL 1,5-DAN in 70% ACN was sprayed onto the tissue sections with the following instrumental parameters: nitrogen flow (12 psi), spray temperature (80 °C), nozzle height (40 mm), five passes with offsets and rotations, and spray velocity (1250 mm/min), and isocratic flow of 50 μL/min using 70% ACN as pushing solvent.

MALDI-IMS was performed on a MALDI TOF/TOF UltrafleXtreme mass spectrometer equipped with SmartBeam II Nd:YAG/355 nm laser. Lipid imaging was performed in reflection negative mode with a source accelerating voltage of − 20 kV. A mass range of 200–2500 Da was analyzed with 20 laser shots per pixel at a spatial resolution of 15 μm with laser focus set to minimum. The mass resolution at *m*/*z* 800 was of M/ΔM 20000. External calibration was carried out using peptide calibration standard I (Bruker Daltonics).

### Data Processing

IMS data processing was done using in house-developed scripts in MATLAB R2018b with Bioinformatics Toolbox 4.11 and Image Processing Toolbox 10.3 (MathWorks, Inc.) installed. MALDI imaging files were converted in FlexImaging version 3.0 software (Bruker Daltonics) from *.mis format to Analyze7.5 format before importing them into MATLAB. The imaging raw data were reshaped into two-dimensional arrays where each row represents a spectrum. Intensity transformations such as logarithms and the square root can be used as variance-stabilizing transformations. In here, we examined log_10_, log_e_ (ln), and square root transformations along with normalization of the raw and transformed data using common normalization methods including total ion current (TIC), median, mean root mean square (RMS), and normalization to the maximum peak [8, 9]. Data arrays prepared for analysis were concatenated with a pixel coordinate matrix and written to a tab-delimited text file with *m*/*z* values in the abscissa header information. The pixel coordinates lend themselves as unique observation identifiers required for subsequent chemometric modeling.

### Multivariate Modeling and Data Analysis

Multivariate statistical modeling was performed in SIMCA version 15.0.2 (Sartorius Stedim Biotech, Umeå, Sweden) and included PCA and OPLS-DA [17, 18]. Data were mean centered but not scaled unless otherwise stated. The number of evaluated components was based on the predictive performance (*Q*^2^) as determined by SIMCA’s seven-block cross-validation [19]. To visualize the PCA and OPLS score images of the individual components, the scores matrixes were transferred to MATLAB and reshaped to reorganize the score values into the coordinate system of the original image. This procedure generated score images of the analyzed sample surface, where scores values can be represented by false-color intensity. The corresponding loadings for the interpretation of the scores were obtained from the SIMCA software directly. Segmentation of cerebellar anatomical regions of interest was achieved through region-based active contour segmentation based on the Chan-Vese algorithm [20]. The segmentation was performed on the PCA score matrix calculated from ln-transformed data. Initial contour localization was provided through rough outlines of the ROIs. Segmentation maps were evolved in 5–50 iterations, and refined with a smoothing factor of 0.1–0.3 and hole-filling. Ambiguous and unassigned pixels were treated as observations with missing class assignment. The unique binary ROI maps were used to subset datasets for variance calculations, and reshaped into the categorical class information matrix (Y-block) for OPLS-DA modeling. The terms *R*^2^X and *R*^2^Y signify the cumulative explained fraction of variation in the X-block and Y-block, respectively. *Q*^2^Y refers to the cumulative predicted fraction of variation in the Y-block, according to cross-validation.

## Results and Discussion

Multivariate image analysis has been widely accepted as first step for unbiased interrogation of complex, chemical imaging data generated with imaging mass spectrometry.

A general issue when evaluating complex IMS data is the technical variance. This variance complicates the robust and sensitive identification of ROI and the quantitative analysis of the ROI-associated MS peak data. Therefore, appropriate IMS data preprocessing is required for robust biological interpretation and the recovery of truncated biologically meaningful information including such of potentially small magnitude but might be biologically relevant (e.g., biomarkers). Further, this highlights the need for objective approaches to compare different processing methods and to evaluate how they may affect image data analysis and to outline relevant ROI. We, therefore, set out to develop a chemometric strategy based on multivariate data analysis workflows for evaluating different IMS data processing methods and their suitability for robust and sensitive image segmentation and ROI annotation as well as to quantify the chemical information associated with histological regions of interest.

**Figure 1:**
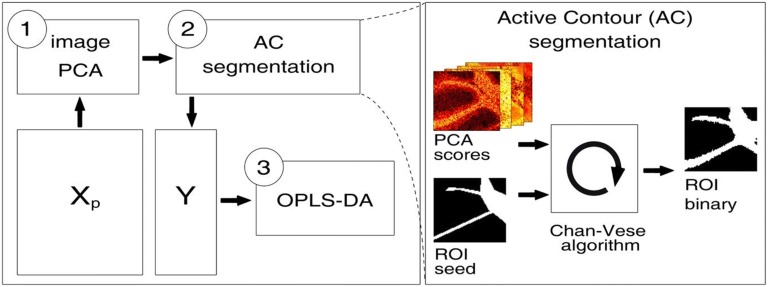
Schematic outline of the chemometric strategy for sensitive ROI annotation and the evaluation of IMS data processing procedures. **(1)** Image PCA is performed to capture the chemical variance in the processed IMS datasets (X_p_). **(2)** Generated PCA score images are subjected to region-based active contour (AC) segmentation for the assignment of anatomical features (ROI) into a Y-block, i.e., generation of class assignment. **(3)** The predictive performances of OPLS-DA models, based on the X- and Y-blocks, are then used for the objective evaluation of the data processing methods applied in X_p_

**Figure 2:**
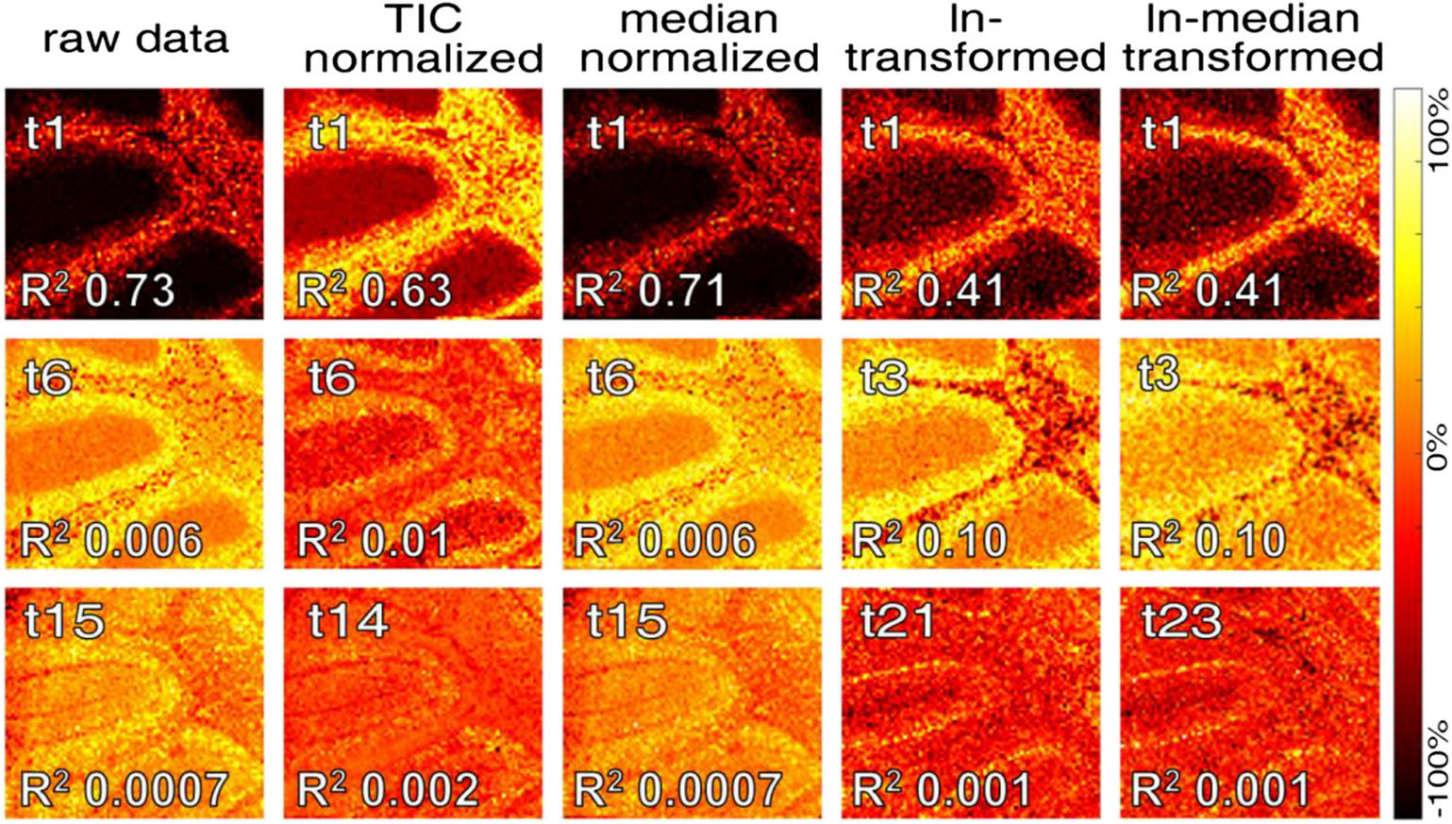
Selected PCA score (*t*_*n*_) images of the MALDI imaging dataset treated with different data processing methods highlight the significant differences regarding feature representation; further PCA score images including those of replicates are provided in the supporting information (Supporting Information Fig. S1-S3), area size 1.26 × 1.13 mm

**Figure 3:**
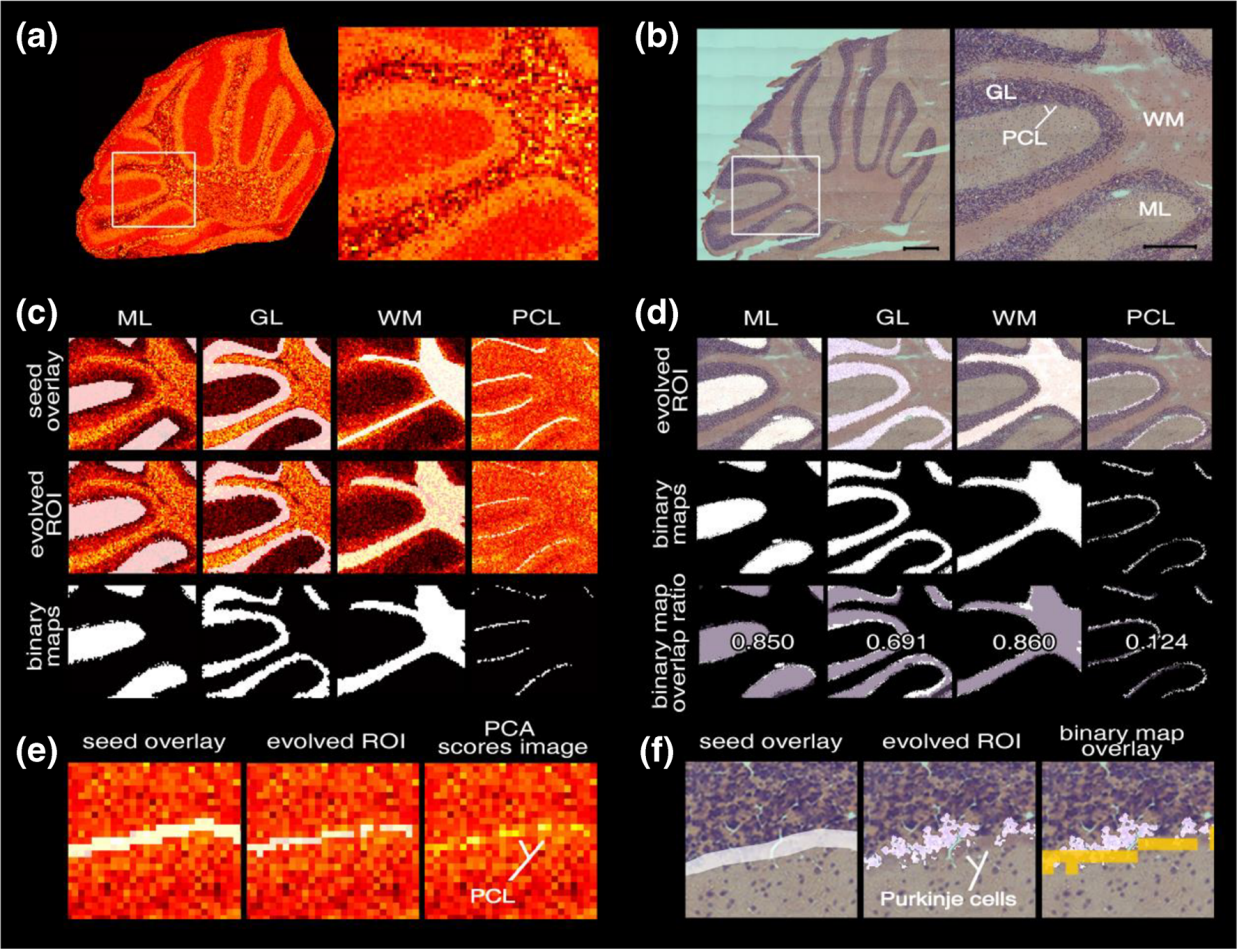
Active contour (AC) segmentation of imaging data into anatomical ROI in mouse cerebellum. (**a**) PCA score image of the entire MALDI IMS-acquired tissue area and the investigated subset, respectively. The white square marks the region of the zoomed view to the right. (**b**) Bright field microscopy image of the H&E-stained tissue section post IMS acquisition. Full view scale bar = 500 μm, zoomed view scale bar = 250 μm, area size 1.26 × 1.13 mm. (**c**) AC segmentation in the PCA scores domain: seed (i.e., initial localization information) for each ROI that was supplied to the AC segmentation algorithm to obtain the evolved ROI, i.e., binary maps after segmentation iterations. (**d**) Binary maps from AC segmentation of the microscopy image and overlays of binary maps of the microscopy image (white) and IMS data (pink), respectively, including their overlap ratio. (**e**) Detailed view to highlight the accuracy of AC segmentation based on the PCA scores matrix and (**f**) the issues arising in the microscopy image regarding PCL annotation; the orange overlay corresponds to the evolved binary map of PCL based on the IMS data

We established an objective, comparative approach based on multivariate modeling to probe the effect of different IMS data processing methods, including transformation and normalization. As an initial step, we investigated the different processing methods on lipid imaging data retrieved from MALDI imaging on mouse cerebellum using image PCA (Figures 1 and 2). We further expanded this approach to employ computational methods for ROI segmentation, including active contour (AC) segmentation (Figures 1 and 3). Finally, we evaluated different normalization approaches, and the combination of different transformation and normalization techniques by multivariate prediction models for the segmented regions of interest.

### PCA to Capture the Informative Variance

For the following study, we acquired MALDI imaging MS data of negative lipid species from mouse cerebellum that is outlined by prominent anatomical ROIs including white matter (WM), molecular layer (ML), granular layer (GL), and Purkinje cell layer (PCL). As a first step following data processing of IMS raw data using the various processing methods, we performed image PCA-based multivariate analysis to evaluate the variance in the IMS dataset pre- and post-processing in an unbiased way. The evaluated data processing methods included transformations and normalization methods. Transformation operations included logarithmic (log, ln) and square root (sqrt) transformations, while normalization approaches included normalization to the total ion current (TIC), normalization to the median, normalization to the mean, root mean square normalization (RMS), and normalization to the maximum peak [8, 9]. Further, the combination of different transformation and normalization techniques was also investigated. Image PCA allowed us to interrogate and visualize variances that are associated with anatomical structures.

When interrogating complex IMS data using multivariate tools such as PCA, it is important to capture a maximum of biological and biochemical variance, respectively, as this might reveal differences in spatial concentration changes that are of histopathological importance. This biological variance associated with anatomical features of interest might, however, be significantly affected by the technical variation across the tissue sample. This technical variation, in turn, will negatively affect accurate ROI segmentation and quantification of the associated chemical information encoded in the mass spectral data [21].

Inspection of the most prominent PCA score images generated for each PCA of differentially processed IMS data revealed significant differences with respect to feature abundance. In detail, all processing and normalization approaches enabled to outline the ML and WM (Figure 2a) as well as the GL regions (Figure 2b) constituting the most prominent anatomical ROI of the cerebellum. However, based on the processing method, these features where detected with varying degree of contrast and prominence. A comparison of the different score images is challenging and difficult to quantify as the most prominent regions were outlined with all processing approaches as well as in the raw data. However, detection of these prominent regions in lower components can serve as one indication for how various pre-processing methods stabilize variance and improve data quality as further reflected in the captured variance within these respective components as indicated by the *R*^2^ value (Figure 2). Here, for median and TIC-normalized data as well as in the raw data, the granular layer is captured in component *t*6 with a *R*^2^ value ≤ 0.01. In contrast, the region is captured in score *t*3 with higher *R*^2^ (0.1) using ln and ln-median transformation (Figure 2b).

In addition, the most significant difference revealed by the different processing methods was the detection of low abundant histological features, such as the PCL. Here, ln and ln-median transformation captured the PCL exclusively, while this was not achieved by the other processing approaches, where the PCL was solely captured along with other features (Figure 2c). Here, ln and ln-median transformation gave the best contrast and allowed to sensitively outline the Purkinje cell layer from the molecular layer and granular layer (Figure 2c, ln: *t*21; ln-median: *t*23; and replicates in Supporting Information Fig. S-2c, ln: *t*21; and Supporting Information Fig. S-3c, ln: *t*16). These results highlight that ln transformation has a significant impact on minimizing inherent data variance allowing sensitive ROI detection.

### Spatial ROI Annotation Using Active Contour Segmentation

Inspection of the PCA score images allows outlining anatomical regions of interest. Here, we show that different processing methods mitigate inherent technical variance to varying extent and in turn allow detection of the various ROI. A small number of PCA score images may be appreciated visually. However, an objective evaluation of data processing methods as well as comprehensive data mining to extract information from data demands an approach that minimizes subjective bias and maximizes information gain and recovery. Therefore, we developed a strategy to investigate how the anatomical ROIs are represented by differently processed data in terms of technical variance and their potential in predictive multivariate modeling. In order to enable the downstream application of supervised multivariate techniques, we set out to partition the dataset into anatomically meaningful ROI, i.e., segmentation, for categorical class assignment corresponding to the generation of a Y-matrix. The majority of methods used for IMS data segmentation are clustering-based methods often performed in the PCA score domain [22–26]. In cluster analysis, data are grouped into clusters based on their similarity in the feature space (e.g., by Euclidean distance). However, common clustering methods disregard a potential spatial coherence of the observations (i.e. pixels) in the dataset as present in image data [27–29]. Furthermore, cluster analysis generates clusters also in uniform or unimodal distributed datasets, where no clusters are expected [30]. Also, for noisy images or images with low contrast, it may be challenging for cluster analysis to achieve a meaningful and anatomically accurate segmentation. And thereby, the presence of significant but low abundant features may be missed [31, 32].

Therefore, for the segmentation of the imaging data in the present study, we applied region-based active contour (AC) segmentation in the PCA score domain. Region-based AC segmentation is an iterative segmentation method based on the Chan-Vese algorithm [20]. Thereby, an initially provided contour (i.e., localization information) is evolved into the segmentation of the proximal region in an iterative process (Supplemental Information Movie 1). The segmentation is unbiased with respect to shrinking or expanding and is not dependent on edge functions to limit the evolution at the desired ROI boundaries. This allows to detect ROI even in very noisy images, and whose boundaries are not necessarily defined by gradient or are very smooth [20]. The AC segmentation was applied for ROI definition on the PCA scores matrix ([*t*_1_, *t*_2_, ..., *t*_*n*_]) of ln-transformed data chosen for its clarity and richness in desired features (Figure 3a). In addition, we performed AC segmentation for ROI definition on brightfield microscopy images of hematoxylin/eosin (HE) stainings obtained from the same tissue section (Figure 3b). For both PCA and HE images, the ROI binary maps generated with AC segmentation allowed the categorical assignment of the original IMS spectra, i.e., pixels to distinct ROI classes (Figure 3c, d). For AC segmentation on PCA score images, accurate ROI binary maps pertaining to the four investigated anatomical features of interest (WM, GL, ML, PCL) were generated (Figure 3c).

Further, AC segmentation of the H&E microscopy images allowed the segmentation of the major anatomical regions (i.e., ML, GL, and WM) with the resulting binary maps being in good agreement with the binary maps obtained through segmentation based on the PCA score matrix as expressed by overlap ratio (Figure 3d, Supporting Information Fig. S4a,b).

However, segmentation of the PCL, as achieved for segmentation of the PCA, on the microscopy image of H&E-stained tissue was not successful (Figure 3e, f). This is explained that the information within the microscopy image is provided by its three color channels only. In contrast, the PCA score matrix provides the linear combinations of the entire biochemical information within the IMS dataset, resulting in a significant increase in chemical contrast. This in turn allows for detection and definition of small histological features such as the PCL which further highlights the potential of IMS for molecular histology.

### Minimizing Non-informative Variance by Logarithmic Transformation

Mass spectral data from MALDI IMS analysis of biological tissues are subject to non-informative variation due to different factors not related to biological or biochemical variation. Variation that affects the imaging data globally can arise from ionization and matrix effects and is commonly remedied by normalization of the spectral intensities [8]. However, IMS datasets also contain variation in form of heteroscedastic noise whereby the variance increases with increasing signal intensity. Thus, signals with higher mean intensities also exhibit larger variances. Datasets with a heteroscedastic error structure are not suitable for statistical modeling with multivariate techniques, as proper modeling requires a consistent noise structure across the entire intensity range. A strategy to alleviate the effects of heteroscedastic noise was previously demonstrated [33, 34]. Here, a variance-stabilizing normalization based on logarithmic transformation was demonstrated to significantly improve the stability of peak intensities [33, 34].

In the present study, we adopted this strategy and examined the variance-stabilizing effect of logarithmic (log, ln) and square root (sqrt) transformations as well as the combination of these transformations with normalization methods. Examination of the noise structure requires data to be of the same class, i.e., representing the same chemical profiles. Therefore, we subdivided the dataset into anatomical ROI using AC segmentation as outlined above and extracted the majority of pixels of each ROI, rather than selecting a smaller subset manually.

**Figure 4:**
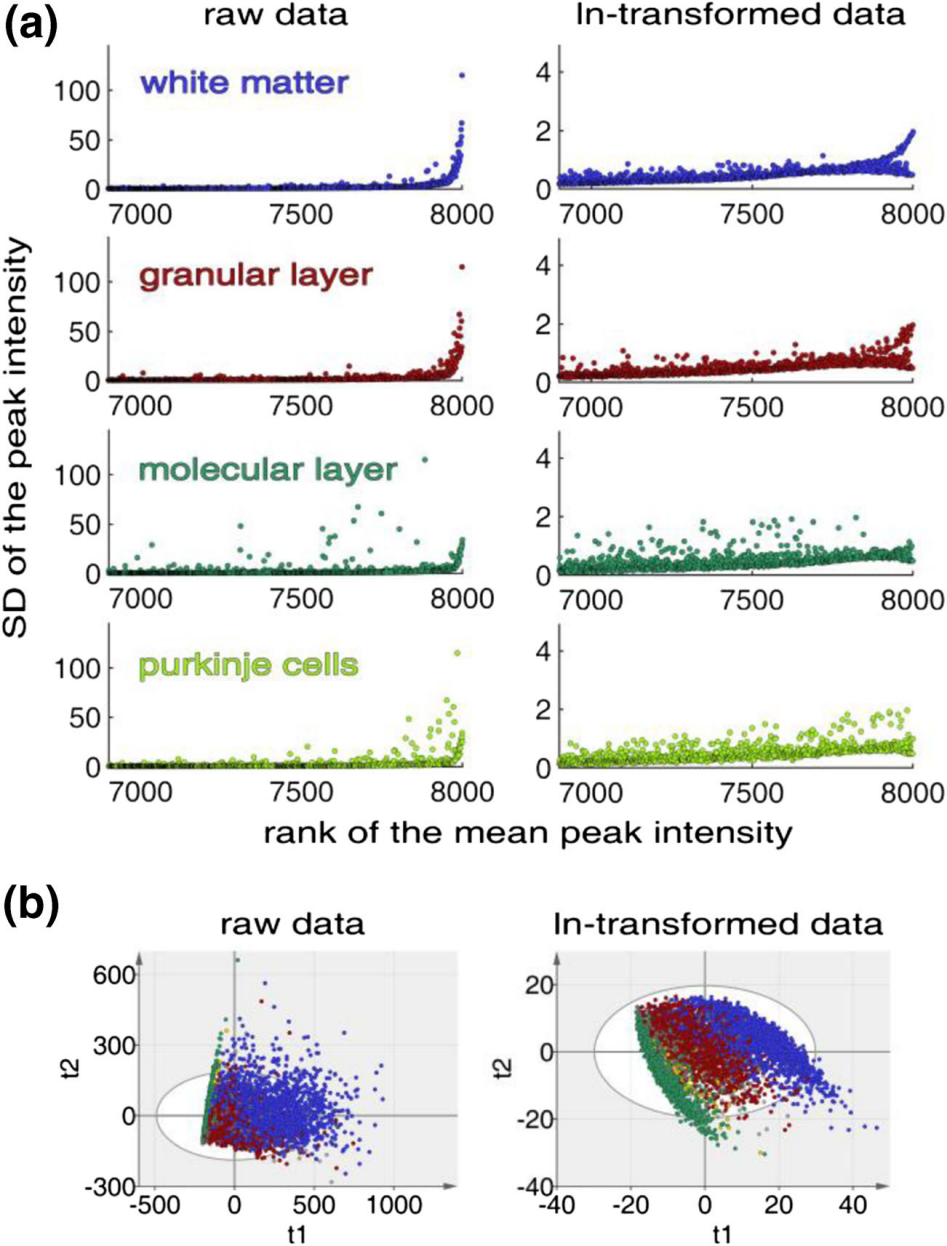
The effect of ln transformation as variance stabilizing transformation. (**a**) Scatter plots to illustrate the heteroscedastic data structure and the effect of ln transformation on the various cerebellar ROI. (**b**) PCA score plots to illustrate the decreased spread and the decreased number of observations outside the 95% confidence interval (oval) and improved clustering after ln transformation of the data

On the scatter plots illustrated in Figure 4a, the standard deviations (SD, *σ*) of each peak were sorted after mean peak intensity. Here, the raw data displayed higher variances (*σ*^2^) for peaks with higher mean peak intensities, i.e., a heteroscedastic noise structure. Logarithmic transformation (ln) of the raw data stabilized the variance in each of the anatomical regions. Furthermore, the transformation improved the clustering on the PCA score plot with a decreased number of observations outside the 95% confidence interval (Figure 4b). Taken together, image segmentation and examination of the ROI-specific noise structure allows the selection of pertinent processing methods for each ROI individually.

### Multivariate Modeling (OPLS-DA) for Qualitative and Quantitative Evaluation of IMS Data Processing Methods

Based on the results showing the impact on normalization and transformation on technical variance, it is of interest to evaluate the different data processing methods on ROI detection and image segmentation. However, an objective measure and quality metric, respectively, for comparing the performance of processing methods for IMS data has not been previously reported. Our aim was, therefore, to establish a chemometric strategy for the objective evaluation of IMS data processing methods for multivariate image analysis in particular.

The strategy is based on the evaluation of the descriptive and predictive abilities (*Q*^2^) of OPLS-DA models built with the processed data. The eventual aim was then to identify the ROI-associated biochemical information conveyed in the best performing model.

**Figure 5:**
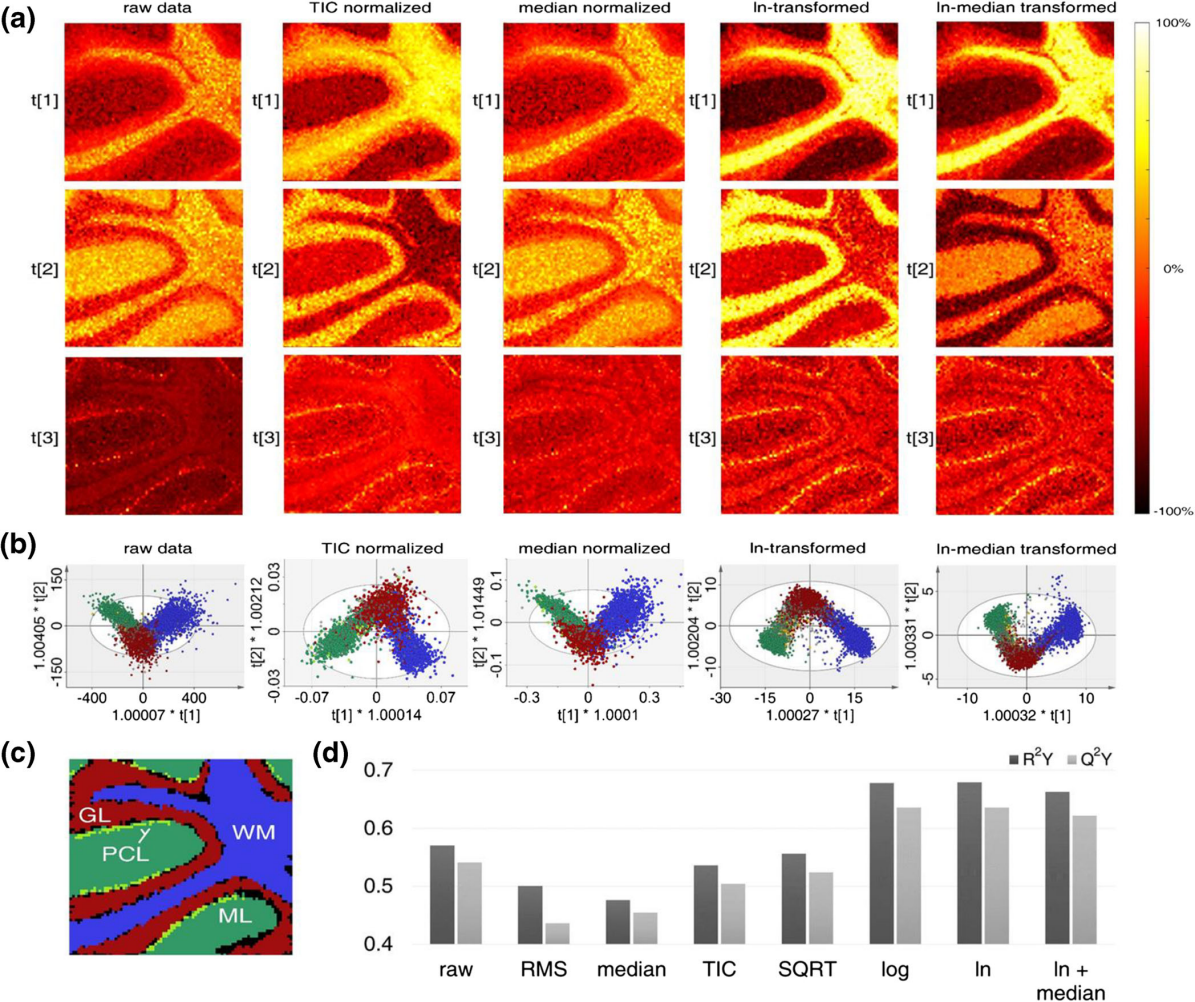
OPLS-DA modeling results from differentially processed data. (**a**) OPLS-DA scores images, area size 1.26 × 1.13 mm. (**b**) OPLS-DA score plots with 95% confidence interval (oval). (**c**) Multicolor map of class assignments used for OPLS-DA modeling: WM white matter, GL granular layer, ML molecular layer, PCL Purkinje cell layer; black pixels are class-unassigned observations. (**d**) OPLS-DA model quality metrics, *R*^2^Y and *Q*^2^Y, signifying the cumulative explained, respectively, predicted fraction of variation in the Y-block

Following image PCA and AC segmentation of the PCA scores, the ROIs were classified. We then generated and evaluated OPLS-DA models for the differently processed IMS datasets. Here, the major anatomical regions, WM, GL, ML, are projected in clusters on two predictive components (*t*1 and *t*2) for all processing methods (Figure 5a, b). The PCL clusters along the third component (*t*3) and was most prominently explained in ln and ln-median transformed data, while only weakly captured in median-normalized data (Figure 5a, b). Similar results were obtained for the analysis of biological replicates (Supporting Information Fig. S-5 and S-6). When performing OPLS-DA, the third component was initially not automatically included for TIC and median normalization due to an arbitrary cutoff applied automatically by the software on how much *Q*^2^ is increased by including an additional component. However, adjustment of this cutoff is required to balance sensitivity on the one hand and potential overfitting of the model.

The contrast within the score images represents the prominence of the underlying chemical profiles of the individual anatomical regions in the acquired IMS spectra. Pixels with missing class information (Figure 5c) obtained score values calculated based on the similarity of their chemical profiles to class-assigned pixels. We then compared the performance of the different processing methods by evaluating the corresponding quality metrics of the generated OPLS-DA model, including descriptive (*R*^2^X, *R*^2^Y) and predictive characteristics (*Q*^2^X, *Q*^2^Y) (Figure 5d; Supporting Information Fig. S5b and S6b).

Here, we observed across all three independent datasets that ln transformation– and ln-median transformation–yielded OPLS models with the highest cumulative predictive ability reflected in the *Q*^2^ values. This suggests ln transformation and ln-median transformation are the most appropriate processing methods for the dataset in this study.

**Figure 6:**
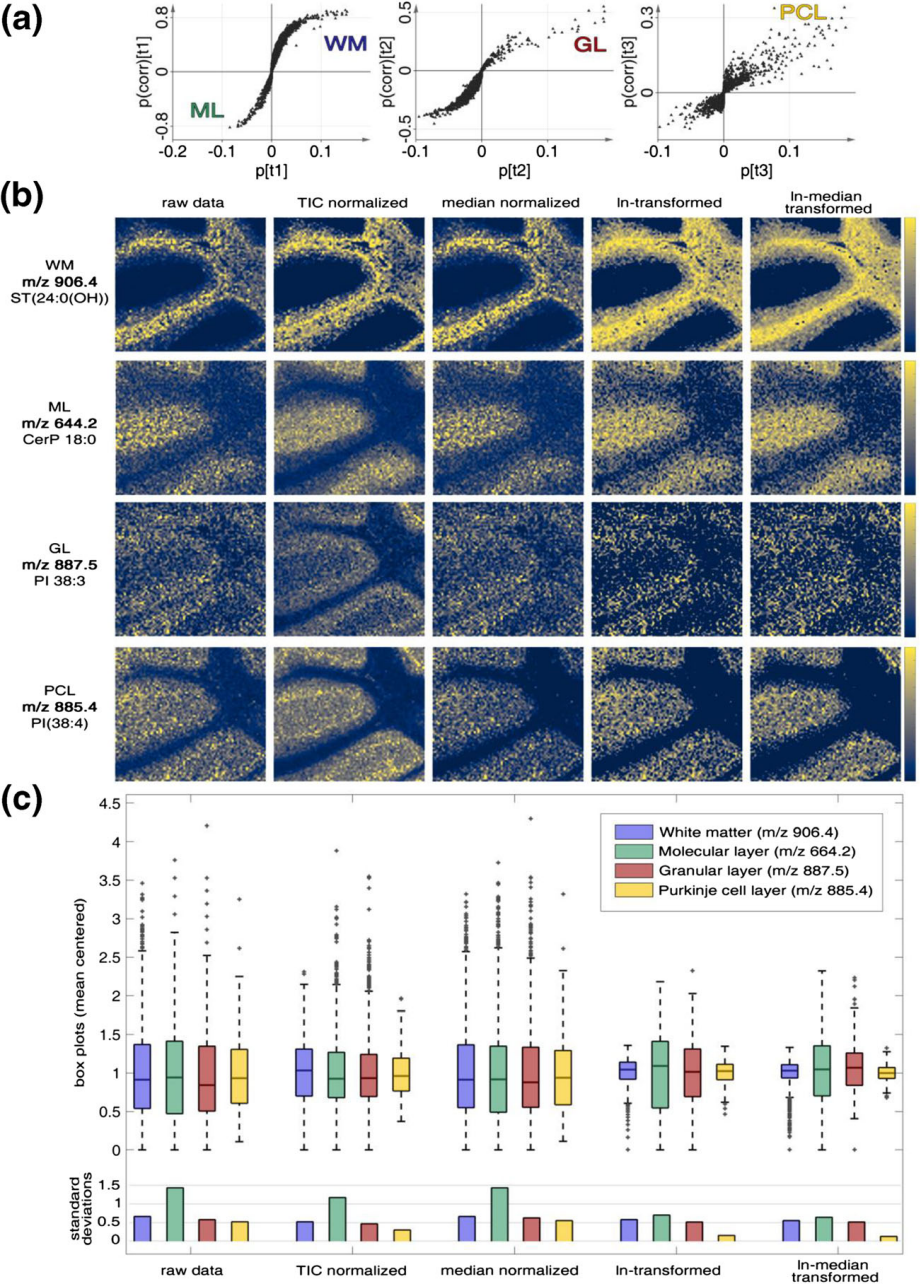
(**a**) S-plots based on OPLS-DA modeling results of ln-transformed data. (**b**) Single ion images of prominent peaks loading for particular cerebellar ROI, area size 1.26 × 1.13 mm, colormap cividis [39]. (**c**) Box plots of mean-centered intensities of ions localizing to the ROI before and after data processing, illustrating spread of the intensity values as well as standard deviation

We then inspected the corresponding loadings of the different OPLS-DA models generated for the different processing methods (Figure 6a). The aim was to identify the chemical correlates associated with the respective anatomical ROI. Here, ML, GL, WM, and PCL were outlined by single ion images of dominant loadings in the corresponding OPLS scores (Figure 6b). In detail, we observed that sulfatide species were the most prominent chemical species associated with the white matter though these species displayed also a strong localization to the granular layer. Sulfatides are constituents of the myelin sheath ensheathing axons which in turn constitute the white matter and innervate the granular layer that constitutes mainly cell bodies of small neurons, i.e., granular cells. In contrast, the molecular layer, as main part of the gray matter, along with the granular and Purkinje cell layers, constitutes mainly of cell bodies. OPLS-DA separated the ML from the WM in *t*1 and the most prominent loadings associated with this separation included ceramide-1-phosphate (CerP) that localized specifically to the ML. This is well in line with previous findings, where higher levels of CerP have been identified for the ML [35–37]. For the GL, OPLS-DA revealed a number of sulfatides that localized prominently to this region as a consequence of the innervating nerve fibers as described previously [36, 37]. Further, distinct phosphoinositol (PI) species, PI 38:3 and PI 38:2, were found to localize to the GL. In contrast, the Purkinje cell layer was distinctively outlined by PI 38:4. This differential localization of PI species with varying fatty acid configuration is well in line with other data, reported previously [38]. These results further support the suitability of the here presented approach to delineate biochemical localization patterns associated with anatomical regions of interest.

For each of the ROI-specific lipid species, we evaluated the effect of the different processing methods, with respect to variance of the signal. Here, we observed that ln and ln-median processing results in a significant decrease in signal variation of all the four lipid species within the respective ROI. This is illustrated in both the spread of the data as well as the relative standard deviation. Here, e.g., for PI 38:4 in the PCL, the RSD decreased from 0.54 to 0.16 and 0.12, upon ln transformation and ln median transformation/normalization, respectively (Figure 6c, replicates in Supporting Information Fig. S-7). These results are in line with the observations on multivariate modeling for ROI detection (PCA) and validation (OPLS-DA), where ln and ln-median processing gave the best performance. Similarly, the single ion intensity statistics (Figure 6c) show that data processing with ln and ln-median presents the best alternative to robustly quantify ROI-specific chemical localization in our IMS data.

## Conclusions

Taken together, we developed a multivariate strategy for robust ROI feature detection, image segmentation, and classification that enabled quantitative comparison of ROI-associated biochemical localization patterns. We further present a region-based, active contour segmentation method that provided accurate segmentation of anatomical regions of interest. We demonstrate that data processing methods have a strong impact on the feature detection and annotation. Here, ln transformation together with median normalization gave the most robust data for feature detection, ROI annotation, and image segmentation and quantification. These results highlight the need to apply appropriate data processing tools for multivariate modeling-based image segmentation in IMS.

## Electronic supplementary material

ESM 1 (DOCX 7677 kb)

ESM 2 (MP4 107,791 kb)
