# Supplementary material for: Chemometric Strategies for Sensitive Annotation and Validation of Anatomical Regions of Interest in Complex Imaging Mass Spectrometry Data
Source: J Am Soc Mass Spectrom. 2019 Sep 16;30(11):2278–88. doi: 10.1007/s13361-019-02327-y (PMC6828630; doi:10.1007/s13361-019-02327-y)
Supplement: Supplementary file 1 — (DOCX 7677 kb) [file 13361_2019_2327_MOESM1_ESM.docx]

Supporting Information

**Chemometric Strategies for Sensitive Annotation and Validation of Anatomical Regions of Interest in Complex Imaging Mass Spectrometry Data**

Patrick M. Wehrli^1^, Wojciech Michno^1^, Kaj Blennow^1,2^, Henrik Zetterberg^1,2,3,4^ and Jörg Hanrieder^1,4^*

1. *Department of Psychiatry and Neurochemistry, Sahlgrenska Academy at the University of Gothenburg, Mölndal, Sweden*
2. *Clinical Neurochemistry Laboratory, Sahlgrenska University Hospital, Mölndal, Sweden*
3. *UK Dementia Research Institute at UCL, London, United Kingdom*
4. *Department of Neurodegenerative Disease, Institute of Neurology, University College London, London, United Kingdom*

*** Contact:**

Jörg Hanrieder, PhD

Dept. Psychiatry and Neurochemistry, Sahlgrenska Academy at the University of Gothenburg, Mölndal Hospital, House V, Biskopsbogatan 27, SE-43180 Mölndal, Sweden

jh@gu.se; +46313432377

Table of content

Figure S-1a-e : PCA scores images, dataset 1, various processing methods

Figure S-2a-c : PCA scores images, dataset 2, various processing methods

Figure S-3a-c : PCA scores images, dataset 3, various processing methods

Figure S-4. Binary maps, dataset 2, dataset 3.

Figure S-5. OPLS-DA modelling results, dataset 2.

Figure S-6. OPLS-DA modelling results, dataset 3.

Figure S-7: Box plots of ion intensities, dataset 2, dataset 3

t1 t2 t3 t4 t5 t6 t7


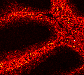

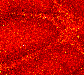

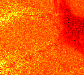

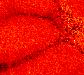

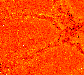

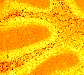

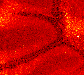


t8 t9 t10 t11 t12 t13 t14


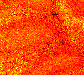

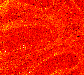

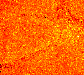

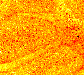

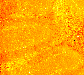

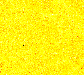

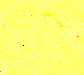


t15 t16 t17 t18 t19 t20 t21


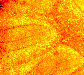

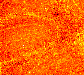

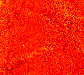

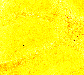

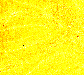

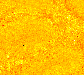

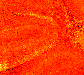


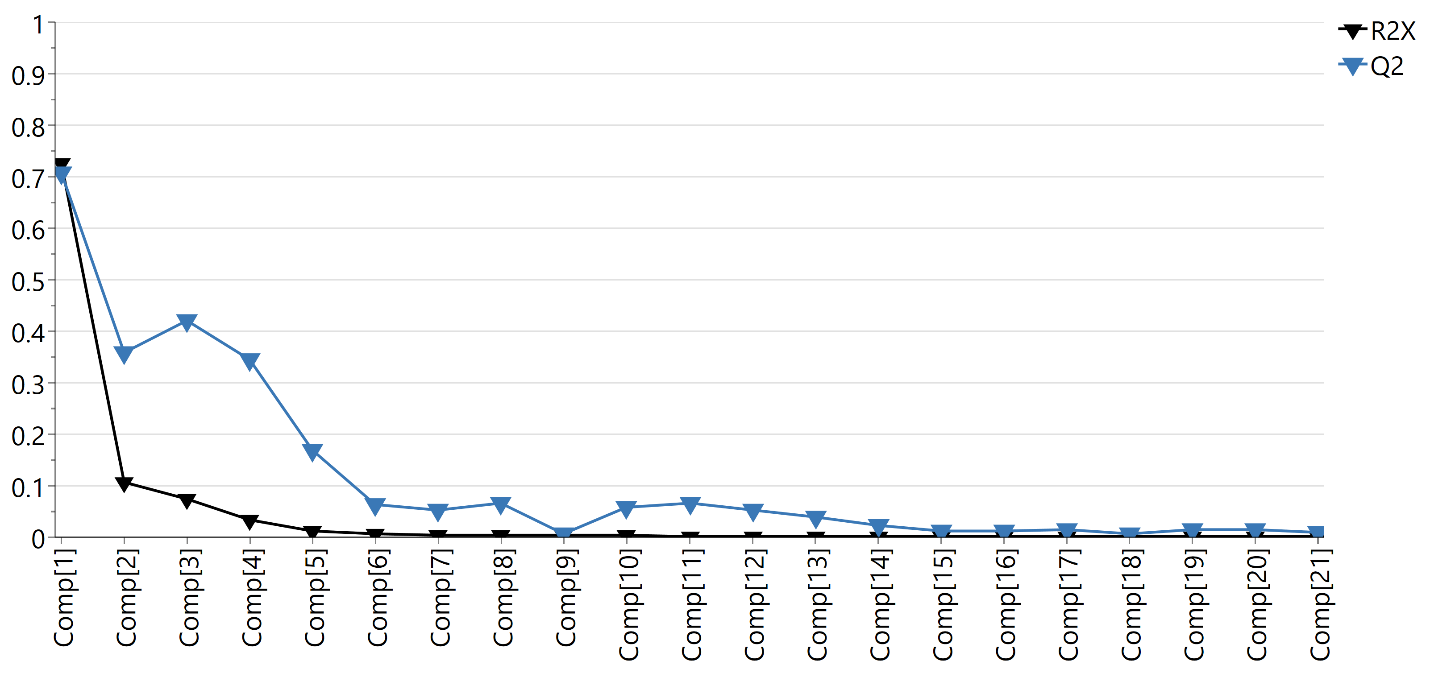


Figure S-1a. PCA scores images and corresponding R^2^ and Q^2^ values, raw data, dataset 1; cumulative R^2^ 0.98, cumulative Q^2^ 0.96.

t1 t2 t3 t4 t5 t6 t7


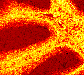

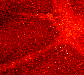

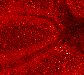

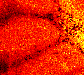

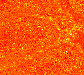

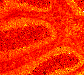

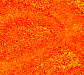


t8 t9 t10 t11 t12 t13 t14


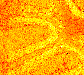

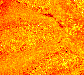

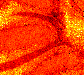

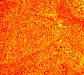

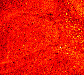

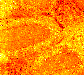

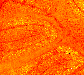


t15 t16 t17 t18 t19 t20


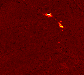

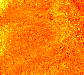

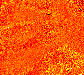

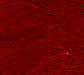

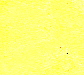

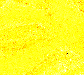


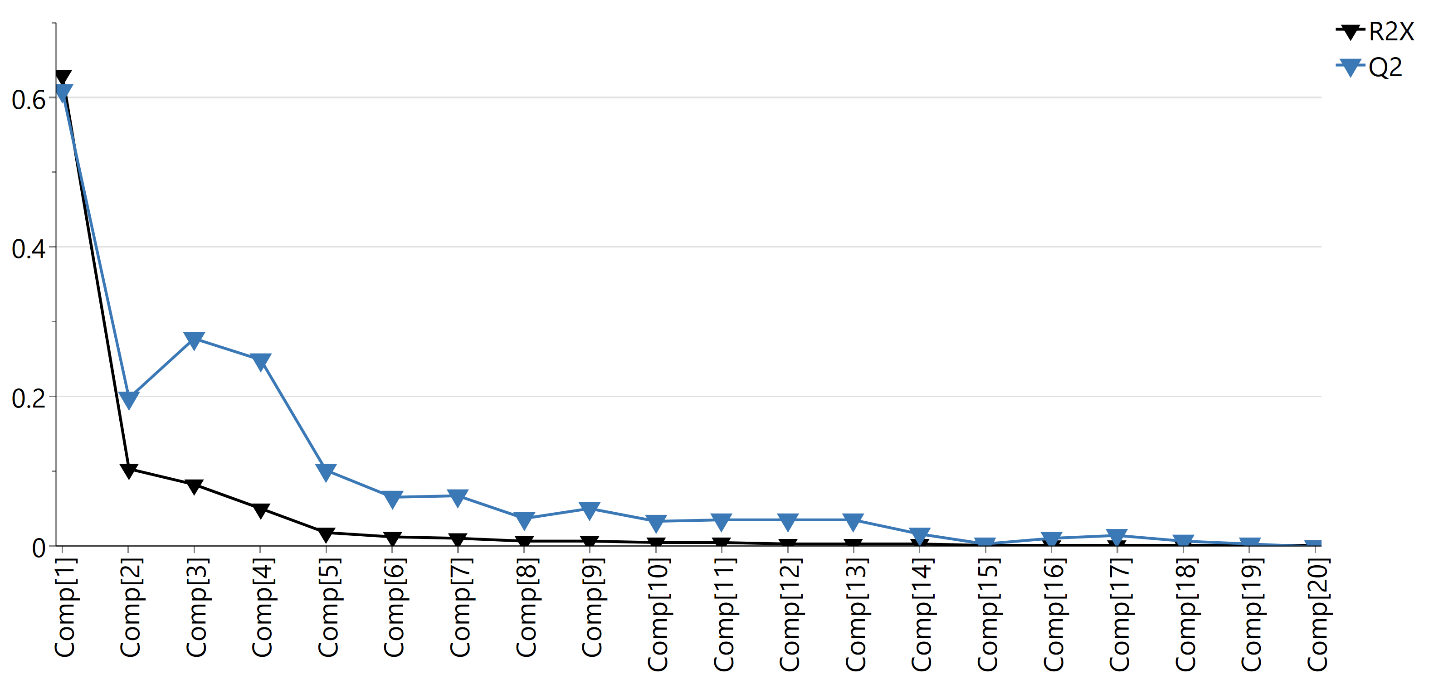


Figure S-1b. PCA scores images and corresponding R^2^ and Q^2^ values, TIC normalized data, dataset 1; cumulative R^2^ 0.95, cumulative Q^2^ 0.90.

t1 t2 t3 t4 t5 t6 t7


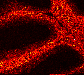

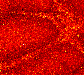

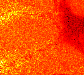

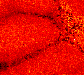

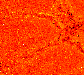

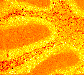

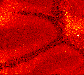


t8 t9 t10 t11 t12 t13 t14


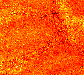

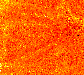

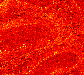

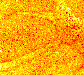

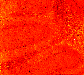

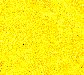

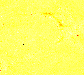


t15 t16 t17 t18 t19 t20 t21


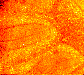

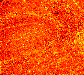

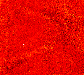

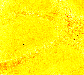

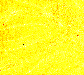

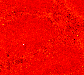

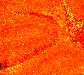


t22 t23 t24 t25 t26


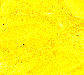

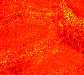

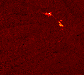

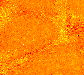

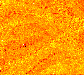


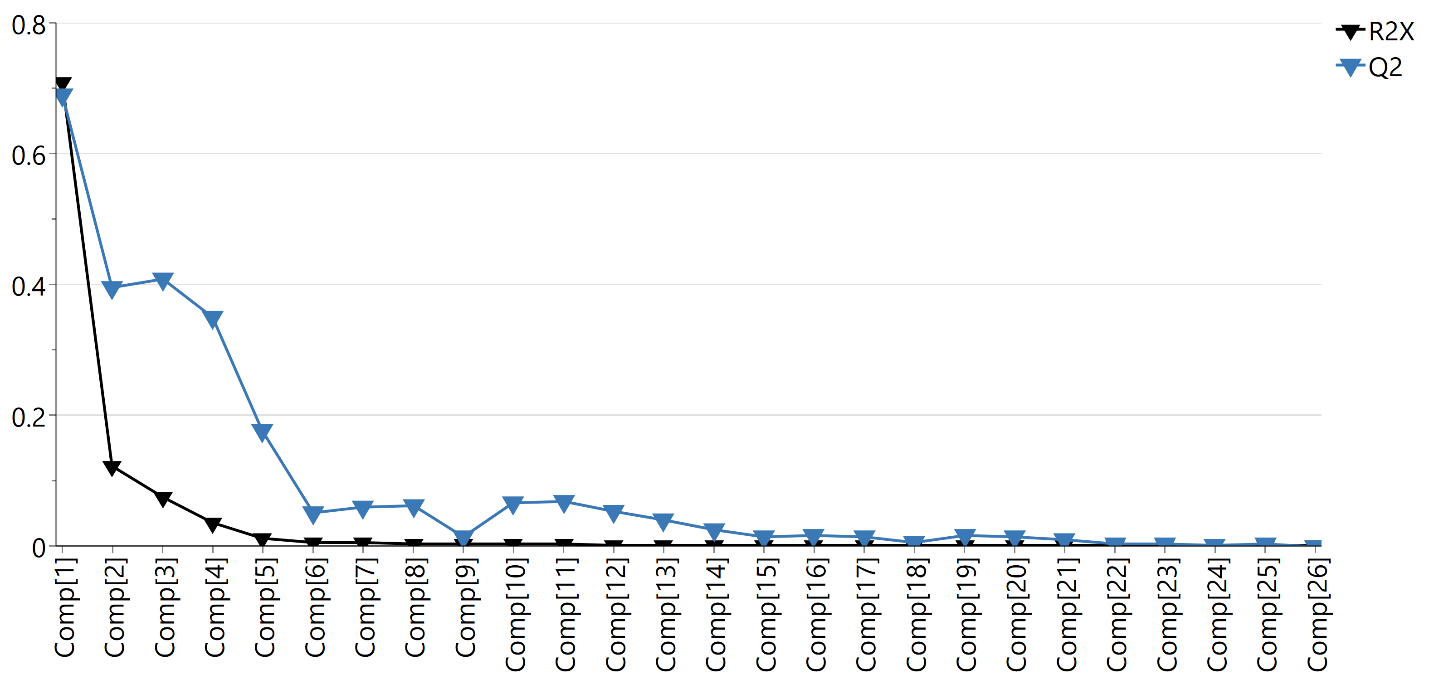


Figure S-1c. PCA scores images and corresponding R^2^ and Q^2^ values, median normalized data, dataset 1; cumulative R^2^ 0.98, cumulative Q^2^ 0.97.

t1 t2 t3 t4 t5 t6 t7


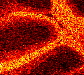

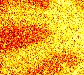

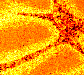

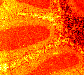

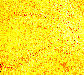

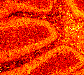

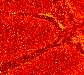


t8 t9 t10 t11 t12 t13 t14


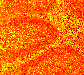

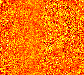

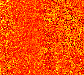

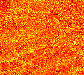

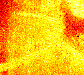

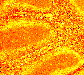

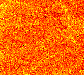


t15 t16 t17 t18 t19 t20 t21


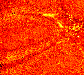

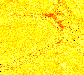

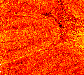

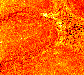

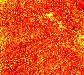

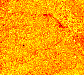

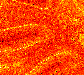


t22 t23 t24


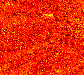

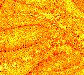

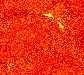


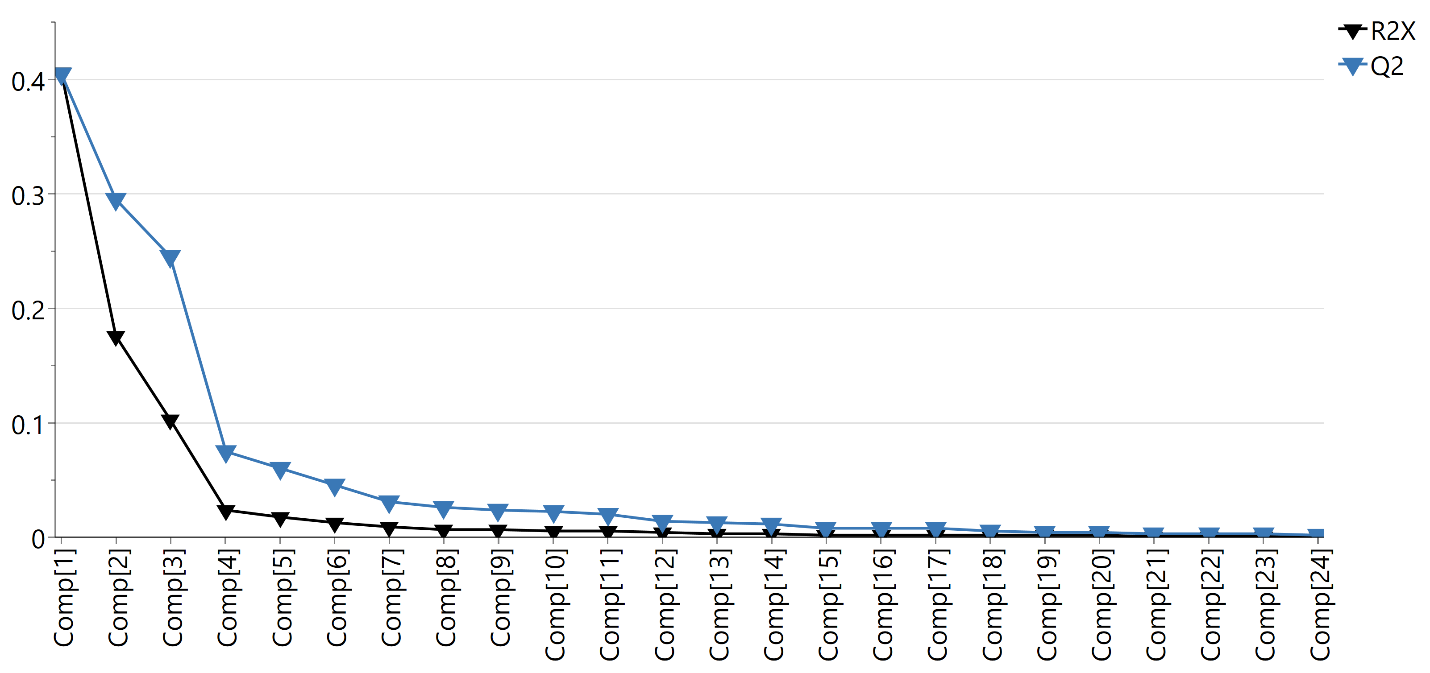


Figure S-1d. PCA scores images and corresponding R^2^ and Q^2^ values, ln-transformed data, dataset 1; cumulative R^2^ 0.80, cumulative Q^2^ 0.79.

t1 t2 t3 t4 t5 t6 t7


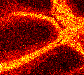

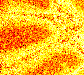

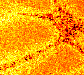

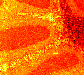

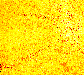


t8 t9 t10 t11 t12 t13 t14

t15 t16 t17 t18 t19 t20 t21

t22 t23 t24 t25 t26

Figure S-1e. PCA scores images and corresponding R^2^ and Q^2^ values, ln-median-processed data, dataset 1; cumulative R^2^ 0.80, cumulative Q^2^ 0.79.

t1 t2 t3 t4 t5 t6

t7 t8 t9 t10 t11 t12

t13 t14 t15 t16

Figure S-2a. PCA scores images and corresponding R^2^ and Q^2^ values, raw data, dataset 2; cumulative R^2^ 0.99, cumulative Q^2^ 0.98.

t1 t2 t3 t4 t5 t6

t7 t8 t9 t10 t11 t12

t13 t14 t15

Figure S-2b. PCA scores images and corresponding R^2^ and Q^2^ values, TIC normalized data, dataset 2; cumulative R^2^ 0.97, cumulative Q^2^ 0.93.

t1 t2 t3 t4 t5 t6

t7 t8 t9 t10 t11 t12

t13 t14 t15 t16 t17 t18

t19 t20 t21 t22 t23 t24

t25 t26 t27

Figure S-2c. PCA scores images and corresponding R^2^ and Q^2^ values, ln-transformed data, dataset 2; cumulative R^2^ 0.84, cumulative Q^2^ 0.81.

t1 t2 t3 t4 t5 t6

t7 t8 t9 t10 t11 t12

t13 t14 t15 t16 t17 t18

t19 t20

Figure S-3a. PCA scores images and corresponding R^2^ and Q^2^ values, raw data, dataset 3; cumulative R^2^ 0.99, cumulative Q^2^ 0.98.

t1 t2 t3 t4 t5 t6

t7 t8 t9 t10 t11 t12

t13 t14 t15 t16 t17

Figure S-3b. PCA scores images and corresponding R^2^ and Q^2^ values, TIC normalized data, dataset 3; cumulative R^2^ 0.97, cumulative Q^2^ 0.94.

t1 t2 t3 t4 t5 t6

t7 t8 t9 t10 t11 t12

t13 t14 t15 t16 t17 t18

t19 t20 t21 t22 t23 t24

t25 t26 t27

Figure S-3c. PCA scores images and corresponding R^2^ and Q^2^ values, ln-transformed data, dataset 3; cumulative R^2^ 0.88, cumulative Q^2^ 0.86.

Figure S-4. Binary maps obtained through AC segmentation for (a) dataset 2 and (b) dataset 3.

Figure S-5. OPLS-DA modelling results from differentially processed data for dataset 2. (a) OPLS-DA scores images; (b) OPLS-DA model quality metrics, R^2^Y and Q^2^Y, signifying the cumulative explained, respectively, predicted fraction of variation in the Y-block.

Figure S-6. OPLS-DA modelling results from differentially processed data for dataset 3. (a) OPLS-DA scores images; (b) OPLS-DA model quality metrics, R^2^Y and Q^2^Y, signifying the cumulative explained, respectively, predicted fraction of variation in the Y-block.

Figure S-7. Box plots of mean centered intensities of ions localizing to the ROI before and after data processing. (a) dataset 2, (b) dataset 3.
